# Clinical significance of the preoperative main pancreatic duct dilation and neutrophil-to-lymphocyte ratio in pancreatic neuroendocrine tumors (PNETs) of the head after curative resection

**DOI:** 10.1186/s12902-019-0454-4

**Published:** 2019-11-12

**Authors:** Bo Zhou, Canyang Zhan, Jie Xiang, Yuan Ding, Sheng Yan

**Affiliations:** 1 Department of Hepatobiliary and Pancreatic Surgery, Second Affiliated Hospital, Zhejiang University School of Medicine, Hangzhou, 310003 China; 2 Department of Neonatology, Children’s Hospital, School of Medicine, Zhejiang University, Hangzhou, China; 3 Department of Hepatobiliary and Pancreatic Surgery, First Affiliated Hospital, School of Medicine, Zhejiang University, Hangzhou, China

**Keywords:** Pancreatic neuroendocrine tumors, Neutrophil-to-lymphocyte ratio, Main pancreatic duct dilation, WHO classification, Prediction

## Abstract

**Background:**

The present study aimed to investigate the prognostic significance of preoperative main pancreatic duct dilation and the neutrophil-to-lymphocyte ratio (PD-NLR) in pancreatic neuroendocrine tumors (PNETs) of the head after curative resection.

**Methods:**

Sixty-four consecutive PNETs of the head that underwent curative resection were included in the study. Preoperative main pancreatic duct dilation (PD) was defined as a pancreatic duct dilation greater than 3 mm before surgery. Patients with both PD and an elevated NLR (> 3.13), with PD or elevated NLR, or neither of these characteristics were allocated a PD-NLR score of 2, 1, or 0, respectively. Univariate, multivariate and Kaplan-Meier analyses were used to calculate overall survival (OS) and disease-free survival (DFS).

**Results:**

Preoperative PD-NLR score was correlated with tumor size (*P* = 0.005), T-stage (*P* = 0.016), lymph node metastasis (*P* <  0.001), distant metastasis (*P* = 0.005), type of hormone production (*P* = 0.006), perineural invasion (*P* = 0.014), and WHO classification (*P* <  0.001). Patients with a high PD-NLR score had a significantly poor OS and DFS relative to those with a low PD-NLR score (*P* <  0.001). In the multivariate analysis, PD-NLR score was an independent predictor of OS and DFS for PNET of the head (both *P* <  0.05). In the analyses of the various subgroups, preoperative PD-NLR score was also a predictor of OS and DFS. Additionally, the survival predictive capability of PD-NLR score was superior to that of WHO classification.

**Conclusions:**

Despite the retrospective nature and small sample size of the present study, the results suggest that preoperative PD-NLR score can serve as an independent prognostic marker of early survival in patients with PNETs of the head undergoing curative resection. Further large prospective studies are necessary to validate our findings.

## Background

Pancreatic neuroendocrine tumors (PNETs) are a heterogeneous group of neoplasms and account for approximately 1–2% of all pancreatic neoplasms and 7.0% of all neuroendocrine tumors [1]. PNETs can be classified as either functional or nonfunctional. The majority of PNETs, ranging from 60 to 90% of all PNETs, are nonfunctional. Complete surgical resection of a PNET has been suggested to be the only potentially curative treatment. In particular, for PNETs localized to the head of the pancreas, resectability criteria are stricter than are those for the body or tail, indicating that an inoperable tumor invades the celiac axis, superior mesenteric artery or the retroperitoneum extensively [2]. However, PNETs are considered more indolent tumors than are tumors of the exocrine pancreas and are associated with better long-term survival rates [3–5]. The clinical course of the disease is usually characterized by an indolent history with a 5-year survival rate exceeding 60% [6].

Several studies have found that multiple host-related factors are associated with survival in PNETs. Intrinsic tumor characteristics used to predict disease progression include tumor size, stage and grade, Ki-67 indices, and lymph node involvement [7–9]. Information regarding these factors is generally useful, but most of these factors are determined only after surgery. Therefore, it is necessary to identify potential prognostic indicators available before surgery. Increasing amounts of evidence suggest that inflammatory cells are an essential component of the tumor microenvironment and play a role in tumor progression [10, 11]. An elevated neutrophil-to-lymphocyte ratio (NLR) has been shown to be correlated with advanced stages and poor prognoses in a variety of human tumors, including PNET [12], hepatocellular carcinoma [13], breast cancer [14], and pancreatic cancer [15]. Moreover, recent studies have reported that the earliest consistent imaging finding of pancreatic cancer is main pancreatic duct dilation [16, 17]. In addition, Gupta N reported an association between final histologic diagnosis of pancreatic malignancy and main pancreatic duct diameter as determined by endoscopic ultrasound [18]. Nanno Y et al. observed that patients with PNET who presented with main pancreatic duct dilation (PD) showed an aggressive clinical course [19].

When considered together, main pancreatic duct dilation and the neutrophil-to-lymphocyte ratio (PD-NLR) may represent a potential predictor of clinical survival. The aim of the present study was to investigate the prognostic significance of PD-NLR score in PNETs of the head after curative resection. Furthermore, we aimed to compare the predictive capability of PD-NLR score for survival with that of other predictive models.

## Methods

### Study population

A total of 64 patients who underwent curative resection for PNET of the head were retrospectively reviewed from September 2002 to July 2016 at the First Affiliated Hospital, Zhejiang University School of Medicine. The diagnosis of PNET was made based on standard histologic criteria. Information on the following characteristics was collected for each patient: pathologic features including tumor size, lymph nodes and stage, gender, surgical approach, symptoms, and patient age. Tumor stage was classified according to the 8th edition of the American Joint Committee on Cancer (AJCC) staging system, and the grade of each PNET was determined based on the 2010 WHO classification of NETs of the GEP system. The retrospective measurement data on the main pancreatic duct were obtained through magnetic resonance cholangiopancreatography (MRCP) or enhanced CT by at least two experienced radiologists. According to the literature, main pancreatic duct dilatation (PD) was defined as a main pancreatic duct with a maximal diameter greater than 3 mm [18, 20]. PD was observed in 19 of 64 patients (29.7%). The NLR was calculated by dividing the absolute neutrophil count by the absolute lymphocyte count. Receiver operating characteristic (ROC) curve analysis showed that the area under the curve (AUC) of NLR was 0.776 and that the optimal cut-off value was 3.13. These values indicated that NLR showed high sensitivity and high specificity in predicting overall survival. As a result, the NLR values were categorized into two groups: ≤ 3.13 and > 3.13. High NLR (> 3.13) was observed in 16 of 64 patients (25%). Patients with both PD and an elevated NLR (> 3.13), PD or an elevated NLR, or neither were allocated a PD-NLR score of 2, 1, or 0, respectively. The study was approved by the Ethics Committee of First Affiliated Hospital of Zhejiang University School of Medicine.

### Follow-up

The patients were monitored after surgery by outpatient visits or telephone calls. Overall survival (OS) was calculated as the time from the date of surgery to the date of death from any cause or the date of last known contact. Disease-free survival (DFS) was calculated as the time from initial diagnosis until relapse. Follow-up was routinely carried out every 6 months for the first 5 years and yearly thereafter. Follow-up examinations included laboratory tests and imaging techniques [12].

### Statistical analysis

All of the statistical analyses were performed using SPSS software, version 16.0 for Windows (SPSS, Chicago, IL, USA). Area under the curve values, obtained from ROC curve analysis, were used to compare the predictive efficacies of PD-NLR score and those of other predictive models. Differences between groups were analysed using Pearson’s chi-square test, Fisher’s exact test or the Mann-Whitney U test as appropriate. Cox proportional hazard models were used to estimate hazard ratios for OS and DFS and to determine independent risk factors. All *p* values were two sided and considered significant when less than 0.05.

## Results

### Patients’ clinicopathological characteristics

Among the 64 patients with PNETs, 29 (45.3%) were men and 35 (54.7%) were women. These patients were diagnosed at a mean age of 53.0 ± 12.17 years and were evaluated over a mean follow-up period of 46.28 ± 38.53 months. The median size of the PNETs was 2.5 (range, 0.8–19.0) cm. Forty-five of 64 patients (70.3%) underwent standard surgical procedures with regional lymph node dissection, including pancreatoduodenectomy (*n* = 43) and total pancreatectomy (*n* = 2). Patients with small tumors (< 2 cm) without any signs of metastasis underwent enucleation (*n* = 19). At the time of the last follow-up visit, 20 patients had relapsed, and 15 patients had died. The numbers of patients classified into grades 1, 2 and 3 were 28, 26 and 10, respectively. The 1-, 3-, and 5-year OS rates were 95, 81, and 67%, respectively, and the 1-, 3-, and 5-year DFS rates were 81, 65, and 65%, respectively.

### Associations of PD, NLR, and PD-NLR score with survival

**Fig. 1:**
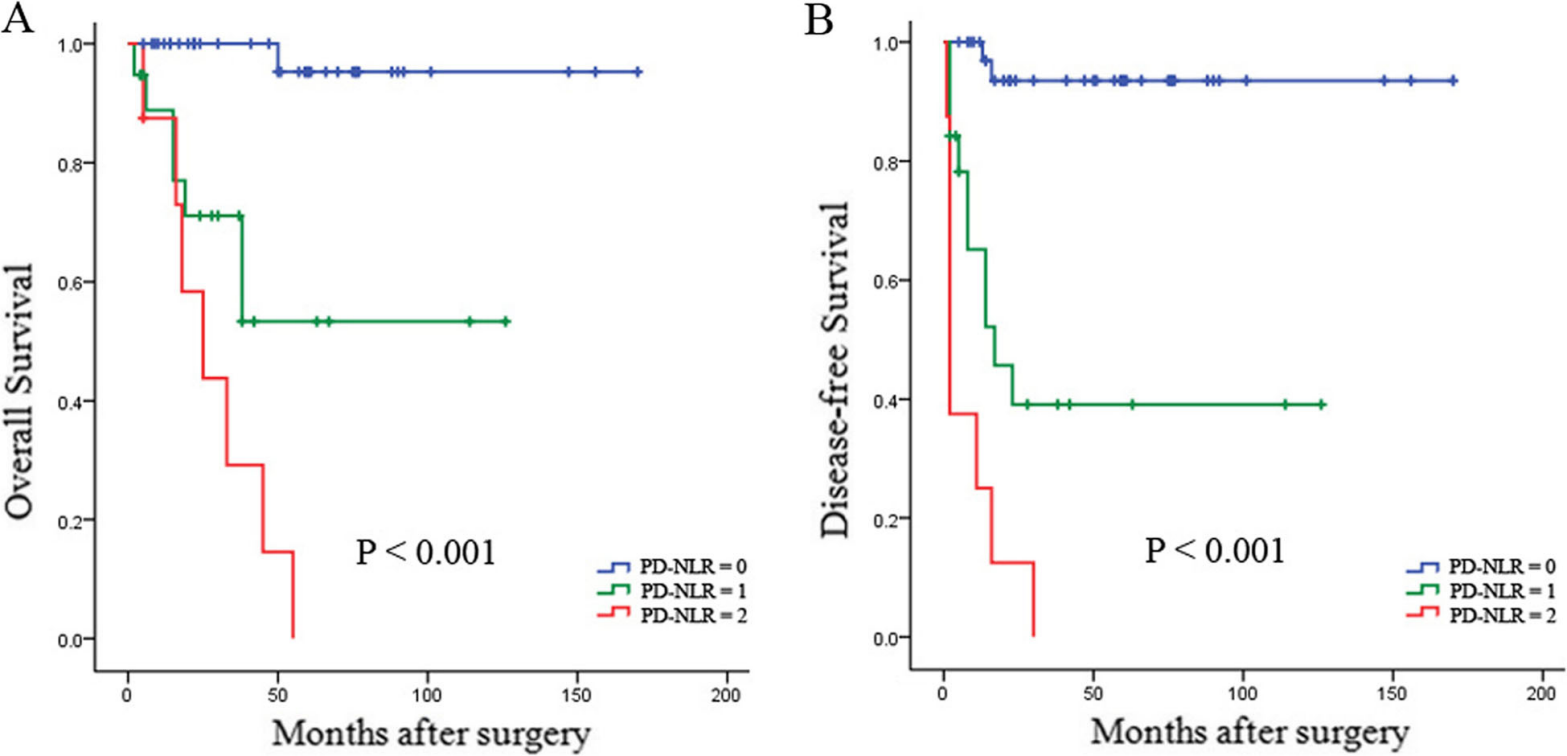
Kaplan-Meier survival curves showing OS (**a**) and DFS (**b**) stratified by PD-NLR in PNET patients undergoing curative resection. High PD-NLR score was significantly correlated with shorter OS and DFS in PNET of the head undergoing curative resection

PD was predictive of inferior OS for PNETs (HR = 9.229, 95% CI 2.879–29.585, *P* <  0.001), whereas patients with a high NLR had shorter OS than patients with a low NLR (HR = 8.837, 95% CI 2.990–26.117, *P* <  0.001). Given the predictive ability of PD and NLR for survival, we analysed the prognostic significance of PD-NLR score. Patients with a PD-NLR score of 1 had shorter OS (HR = 20.055, 95% CI 2.444–164.556, *P* = 0.005) and DFS (HR =14.132, 95% CI 3.083–64.774, *P* = 0.001) than patients with a PD-NLR score of 0 (Fig. 1). Furthermore, a high tumor grade, the presence of lymph node (LN) metastasis and perineural invasion, a large tumor and a high T stage tumor were prognostic factors for poor OS (*P* <  0.05 for all), whereas a symptomatic tumor; a high tumor grade; the presence of LN metastasis, distant metastasis or perineural invasion; a large tumor and a high T stage tumor were associated with poor DFS (*P* <  0.05 for all).

In the multivariate analysis, PD-NLR score remained significantly associated with OS (HR = 16.159, 95% CI 1.902–137.304, *P* = 0.011) and DFS (HR = 7.356, 95% CI 1.495–36.194, *P* = 0.014) (Tables 1 and 2). Furthermore, WHO grade was an independent predictive factor for OS (*P* <  0.05, Table 1), whereas WHO grade and tumor size were independent predictive factors for DFS (*P* <  0.05 for all, Table 2).

**Table 1:** Variables associated with OS according to the Cox proportional hazards regression model

Variable	Univariate analysis			Multivariate analysis		
	HR	95% CI	*P*	HR	95% CI	*P*
Age (years)						
≤ 60	Reference					
> 60	2.074	0.721–5.966	0.176			
Gender						
Female	Reference					
Male	1.064	0.385–2.939	0.905			
Tumor size (cm)						
≤ 2.5	Reference			Reference		
> 2.5	7.336	1.655–32.514	**0.009**	NA	NA	0.249
Symptoms						
Absent	Reference					
Present	3.223	0.727–14.292	0.123			
PD-NLR						
0	Reference			Reference		
1	20.055	2.444–164.556	**0.005**	16.159	1.902–137.304	**0.011**
2	56.819	6.874–469.680	**< 0.001**	32.737	3.561–300.966	**0.002**
Type of hormone production						
Functioning	Reference					
Non-functioning	33.418	0.265–4211.462	0.155			
T-stage						
T1–2	Reference			Reference		
T3–4	3.602	1.303–9.953	**0.013**	NA	NA	0.567
LN metastasis						
Absent	Reference			Reference		
Present	6.896	2.359–20.157	**< 0.001**	NA	NA	0.608
Distant metastasis						
Absent	Reference					
Present	3.438	0.426–27.722	0.246			
Perineural invasion						
Absent	Reference			Reference		
Present	6.453	2.247–18.537	**0.001**	NA	NA	0.118
WHO classification						
Grade 1–2	Reference			Reference		
Grade 3	19.191	4.735–77.784	**< 0.001**	5.222	1.227–22.218	**0.025**

*OS* Overall survival, *PD* Main pancreatic duct dilation, *NLR* Neutrophil-to-lymphocyte ratio, *LN* Lymph node. *P*-values < 0.05, marked in bold font, indicate statistical significance

**Table 2:** Variables associated with DFS according to the Cox proportional hazards regression model

Variable	Univariate analysis			Multivariate analysis		
	HR	95% CI	*P*	HR	95% CI	*P*
Age (years)						
≤ 60	Reference					
> 60	1.321	0.525–3.324	0.554			
Gender						
Female	Reference					
Male	1.481	0.613–3.577	0.382			
tumor size (cm)						
≤ 2.5	Reference			Reference		
> 2.5	7.449	2.177–25.491	**0.001**	4.109	1.054–16.023	**0.042**
Symptoms						
Absent	Reference			Reference		
Present	4.935	1.143–21.302	**0.032**	NA	NA	0.185
PD-NLR						
0	Reference					
1	14.132	3.083–64.774	**0.001**	7.356	1.495–36.194	**0.014**
2	41.928	8.731–201.359	**< 0.001**	15.144	2.762–83.020	**0.002**
Type of hormone production						
Functioning	Reference					
Non-functioning	34.588	0.572–2090.521	0.09			
T-stage						
T1–2	Reference			Reference		
T3–4	2.539	1.034–6.234	**0.042**	NA	NA	0.222
LN metastasis						
Absent	Reference			Reference		
Present	5.789	2.368–14.152	**< 0.001**	NA	NA	0.394
Distant metastasis						
Absent	Reference			Reference		
Present	17.364	4.136–72.889	**< 0.001**	NA	NA	0.271
Perineural invasion						
Absent	Reference			Reference		
Present	4.419	1.681–11.620	**0.003**	NA	NA	0.287
WHO classification						
Grade 1–2	Reference					
Grade 3	14.287	5.321–38.358	**< 0.001**	6.595	1.846–23.565	**0.004**

*DFS* Disease-free survival, *PD* Main pancreatic duct dilation, *NLR* Neutrophil-to-lymphocyte ratio, *LN* Lymph node. *P*-values < 0.05, marked in bold font, indicate statistical significance

### Relationships between PD-NLR and clinicopathological characteristics

Preoperative PD-NLR score was associated with tumor size (*P* = 0.005), T-stage (*P* = 0.016), distant metastasis (*P* = 0.005), LN metastasis (*P* <  0.001), type of hormone production (*P* = 0.006), perineural invasion (*P* = 0.014), and WHO classification (*P* <  0.001). There were no significant associations between preoperative PD-NLR score and the remaining clinicopathological parameters, such as age, gender and symptoms (all *P* > 0.05, Table 3).

**Table 3:** Relationships between PD-NLR and clinicopathological characteristics in patients with surgically resected neuroendocrine tumors in the head of the pancreas

Variable	No. of cases	PD-NLR			*P*
	(*n* = 64)	0 (*n* = 37, %)	1 (n = 19, %)	2 (*n* = 8, %)	
Age (years)					
≤ 60	44	27 (73)	13 (68.4)	4 (50)	0.245
> 60	20	10 (27)	6 (31.6)	4 (50)	
Gender					
Female	35	22 (59.5)	8 (42.1)	5 (62.5)	0.687
Male	29	15 (40.5)	11 (57.9)	3 (37.5)	
tumor size (cm)					
≤ 2.5	33	24 (64.9)	8 (42.1)	1 (12.5)	**0.005**
> 2.5	31	13 (35.1)	11 (57.9)	7 (87.5)	
Symptoms					
Absent	20	14 (37.8)	6 (31.6)	0	0.061
Present	44	23 (62.2)	13 (68.4)	8 (100)	
T-stage					
T1–2	49	32 (86.5)	13 (68.4)	4 (50)	**0.016**
T3–4	15	5 (13.5)	6 (31.6)	4 (50)	
LN metastasis					
Absent	49	34 (91.9)	13 (68.4)	2 (25)	**< 0.001**
Present	15	3 (8.1)	6 (31.6)	6 (75)	
Distant metastasis					
Absent	61	37 (100)	18 (94.7)	6 (75)	**0.005**
Present	3	0	1 (5.3)	2 (25)	
Type of hormone production					
Non-functioning	48	23 (62.2)	17 (89.5)	8 (100)	**0.006**
Functioning	16	14 (37.8)	2 (10.5)	0	
Perineural invasion					
Absent	56	35 (94.6)	16 (84.2)	5 (87.5)	**0.014**
Present	8	2 (5.4)	3 (15.8)	3 (12.5)	
WHO classification					
Grade 1	28	25 (67.6)	3 (15.8)	0	**< 0.001**
Grade 2	26	12 (32.4)	11 (57.9)	3 (37.5)	
Grade 3	10	0	5 (26.3)	5 (62.5)	

*PD* Main pancreatic duct dilation, *NLR* Neutrophil-to-lymphocyte ratio, *LN* Lymph node. *P*-values < 0.05, marked in bold font, indicate statistical significance

### Prognostic value of preoperative PD-NLR in different PNET subgroups

**Fig. 2:**
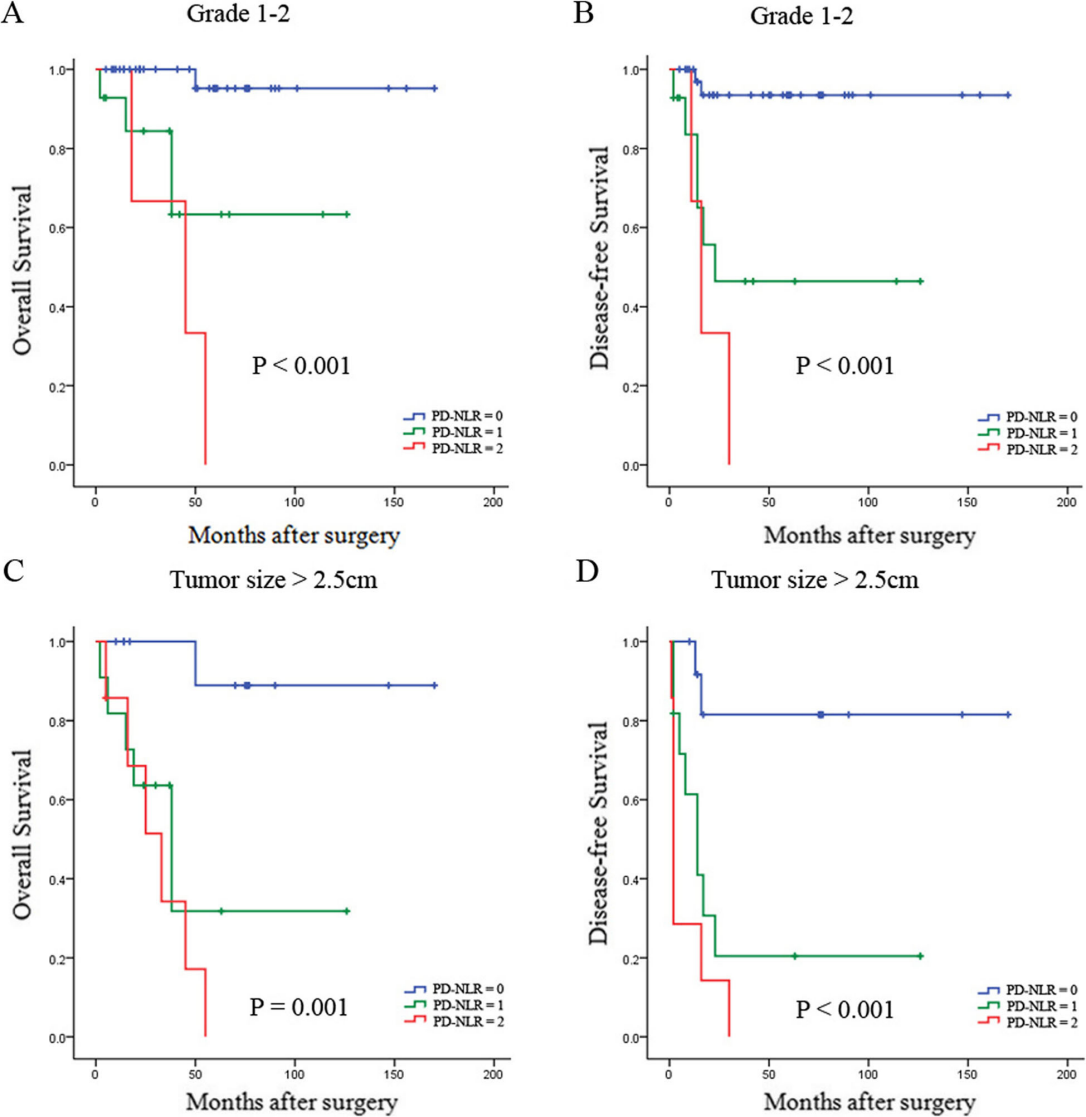
Kaplan-Meier survival curves for the different PNET subgroups. High PD-NLR score was significantly correlated with shorter OS and DFS in subgroups with grade 1–2 (**a** and **b**) and tumor size > 2.5 cm (**c** and **d**)

**Fig. 3:**
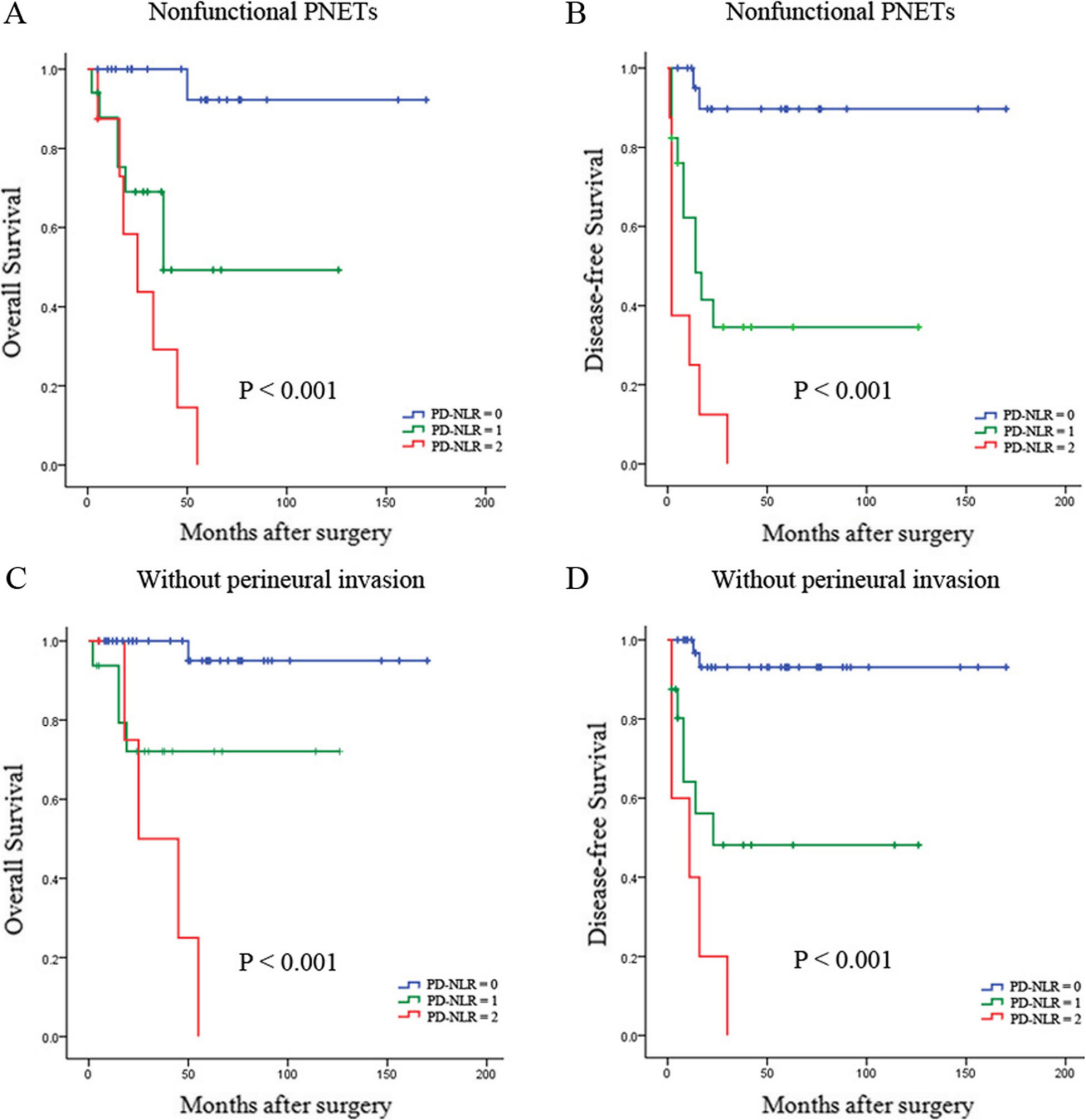
Kaplan-Meier survival curves for the different PNET subgroups. High PD-NLR score was significantly correlated with shorter OS and DFS in subgroups with nonfunctional PNETs (**a** and **b**) and without perineural invasion (**c** and **d**)

As WHO classification, tumor size, type of hormone production and perineural invasion have been identified as prognostic factors for PNET, we next investigated the prognostic value of preoperative PD-NLR score in different subgroups of PNET patients to exclude these factors. Patients with a low PD-NLR (score 0) showed better OS and DFS than those in the high PD-NLR group (score 1 or 2) among patients with grade 1 or 2 tumor (both *P* <  0.001) (Fig. 2a and b). Furthermore, in the patients with tumor size > 2.5 cm, high PD-NLR score was significantly associated with decreased OS (*P* = 0.001) and DFS (*P* <  0.001) (Fig. 2c and d), and the prognostic value of OS (*P* <  0.001) and DFS (*P* <  0.001) was maintained in patients with nonfunctional PNETs (Fig. 3a and b). In addition, high PD-NLR score was a prognostic factor for poor OS and DFS in those patients without perineural invasion (both *P* <  0.001) (Fig. 3c and d).

### Comparative performance of PD-NLR and other predictive models

**Fig. 4:**
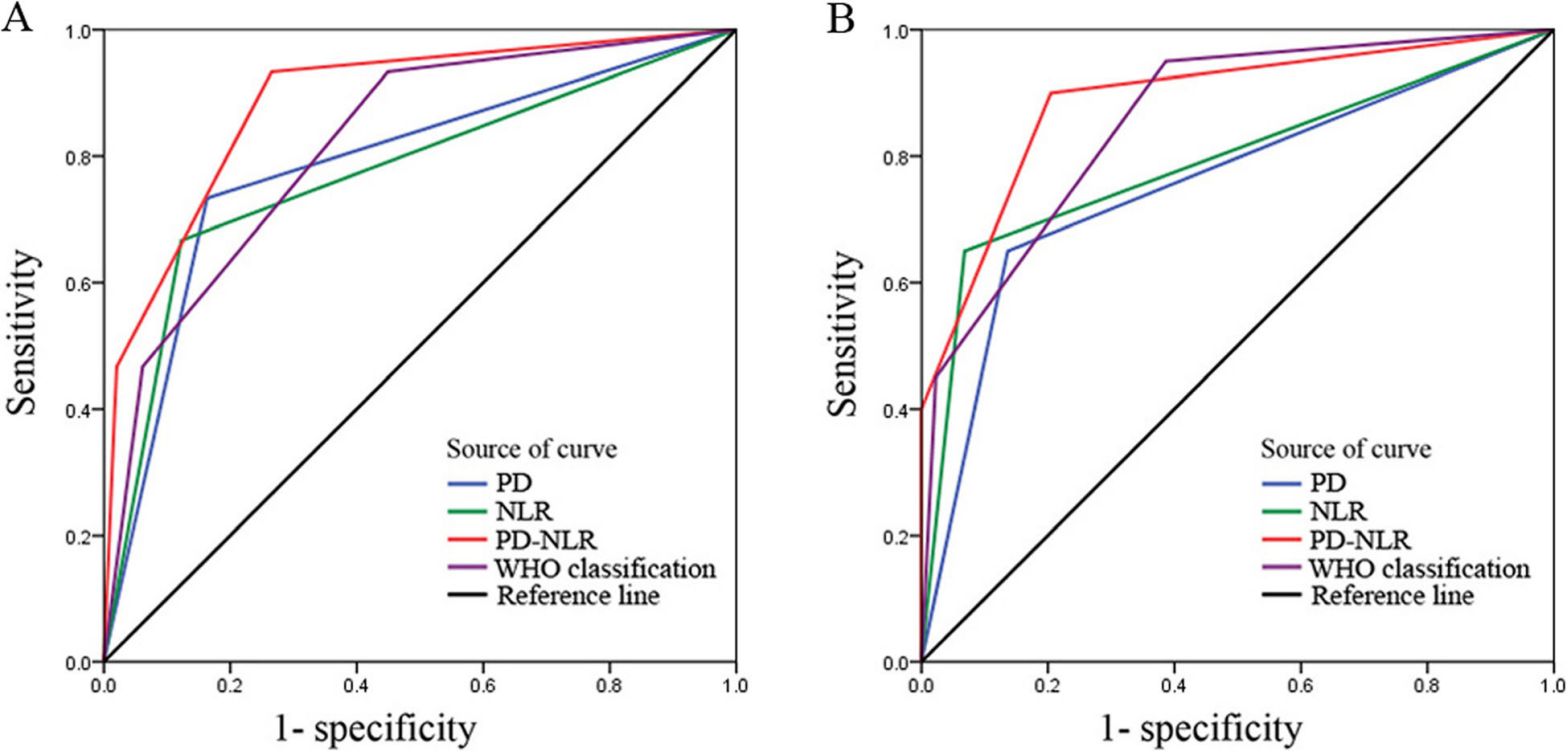
Comparison of the area under the receiver operating characteristic curve (AUC) in different predictive models. The discriminatory capability of PD-NLR was superior to that of other predictive models in OS (**a**) and DFS (**b**) prediction

To further evaluate the prognostic value of PD-NLR, PD, NLR and WHO classification, ROC analysis was performed, and AUC values were compared. PD-NLR had a higher AUC value for OS and DFS (0.886 and 0.889) than PD or NLR (Fig. 4). In addition, the discriminatory capability of PD-NLR was superior to that of the WHO classification in both OS (AUC value: 0.886 vs 0.818) and DFS (AUC value: 0.889 vs 0.858) prediction (Table 4).

**Table 4:** Areas under the ROC curve for WHO classification and PD-NLR for predicting OS and DFS in patients with surgically resected neuroendocrine tumors in the head of the pancreas

Variable	OS		DFS	
	Area under the ROC curve (95% CI)	*P*	Area under the ROC curve (95% CI)	*P*
PD-NLR	0.886 (0.788–0.985)	**< 0.001**	0.889 (0.795–0.983)	**< 0.001**
PD	0.785 (0.641–0.929)	**0.001**	0.757 (0.618–0.896)	**0.001**
NLR (≤ 3.1/ > 3.1)	0.772 (0.619–0.925)	**0.002**	0.791 (0.655–0.927)	**< 0.001**
WHO classification	0.818 (0.699–0.938)	**< 0.001**	0.858 (0.761–0.955)	**< 0.001**

*ROC* Receiver operating characteristic, *OS* Overall survival, *DFS* Disease-free survival, *PD* Main pancreatic duct dilation, *NLR* Neutrophil-to-lymphocyte ratio. *P*-values < 0.05, marked in bold font, indicate statistical significance

## Discussion

The present study showed that preoperative PD-NLR score, a novel and easily accessible inflammation-based score derived from main pancreatic duct dilation and the neutrophil-to-lymphocyte ratio, was an independent prognostic factor for PNET of the head undergoing curative resection. Furthermore, high preoperative PD-NLR score was associated with high tumor grade, large tumor size, LN metastasis, distant metastasis and perineural invasion. In addition, we found that the predictive capability of PD-NLR score was superior to that of other predictive models, including PD, NLR and WHO classification.

Recently, there has been accumulating evidence that PD is associated with a high risk for pancreatic tumors, including pancreatic cancer or PNETs [17–20]. Concerning the mechanism of PD in pancreatic tumors, mechanical compression by the tumor or cancer cell invasion may cause segmental obstruction and upstream dilatation in the main pancreatic duct. Compared with classic PNETs, which have scant stroma and proliferation of medullary pattern, PNETs with PD are more fibrotic and show greater infiltration into adjacent structures, resulting in the entrapment of the main pancreatic duct inside the tumor mass [19, 21, 22]. In addition, the pancreas with PD may be the site where pancreatic cancer originates. Tanaka S et al. reported that main pancreatic duct dilatation (> 2.5 mm) and presence of a pancreatic cyst (> 5 mm) were both strong independent predictors of the subsequent development of pancreatic cancer [23]. Nanno Y et al. found that PNET patients with main pancreatic duct involvement and dilation showed significantly worse recurrence-free survival than did those without ductal involvement (*P* <  0.001), with a 5-year recurrence-free rate of 41% [19]. Therefore, PD can act as a significant prognostic biomarker in pancreatic tumors.

Systemic inflammation is correlated with worse survival in cancer patients in a variety of cancer types [24, 25]. The NLR, which can reflect the systemic inflammation status, has been shown to be a reliable predictive marker for different types of cancer, such as PNET [12], pancreatic ductal adenocarcinoma [15], renal cell carcinoma [26], breast cancer [14] and liver cancer [13]. Salman T et al. revealed that an advanced stage was accompanied by significantly higher NLR and platelet-to-lymphocyte ratio (PLR) in patients with NETs [27]. Recently, Luo G et al. conducted a retrospective analysis of 165 PNETs and reported that NLR was an independent predictor of overall survival for patients with PNETs [28]. Tong Z et al. demonstrated that increased NLR was related with advanced T stage, LN metastasis, tumor thrombus formation, and advanced grade in patients with PNETs [29]. Additionally, our previous study found that NLR and PLR were significantly higher in patients with PNETs than in matched healthy volunteers. Furthermore, NLR, but not PLR, is an independent prognostic factor of both OS and DFS in patients with PNET [30]. In general, preoperative NLR, a prognosis-related serum biomarker, can be useful for predicting disease prognosis in patients with surgically resected PNETs.

In our study, we found that high PD-NLR score was correlated with tumor size, T-stage, lymph node metastasis, type of hormone production, perineural invasion, and WHO classification. Moreover, patients with high PD-NLR score were more likely to have distant metastasis than patients with low PD-NLR score. All of these findings indicate that PD-NLR can reflect the tumor progression and tumor burden. Further analysis revealed that PD-NLR score was an independent predictive factor for patients with surgically resected PNETs of the head. Patients with a high PD-NLR score had significantly poorer OS and DFS than did those with a low PD-NLR score. For the subgroups of patients with grade 1 or 2 tumor or tumor size > 2.5 cm, we similarly found that high PD-NLR score was significantly associated with decreased OS and DFS. Furthermore, the predictive capability of PD-NLR score for survival was superior to that of WHO classification. All of these findings confirm that preoperative PD-NLR score can predict the outcomes of patients with PNET of the head undergoing curative resection.

The major limitations of the present study are its retrospective nature and single-center design. A further limitation is the small sample size. Therefore, large prospective studies are necessary to validate our findings. Another limitation was the short study duration of 46 months, as PNETs generally have an indolent disease course. In addition, only patients who underwent curative resection were included in the study. Despite these limitations, the present study is valuable as it identifies PD-NLR score as a potential prognostic marker to predict survival in patients with PNET of the head undergoing curative resection.

## Conclusions

Based on the PD-NLR scoring system, patients with PNETs of the head after curative resection were classified into three groups based on their prognosis. As a novel and easily accessible inflammation-based biomarker, preoperative PD-NLR score can serve as an independent prognostic marker of early survival in patients with PNETs of the head undergoing curative resection. Prospective and independent studies are warranted to verify our findings.

## Data Availability

All original data are available upon request.

## Abbreviations

AJCC: American Joint Committee on Cancer criteria
DFS: Disease-free survival
MRCP: Magnetic resonance cholangiopancreatography
NLR: Neutrophil-to-lymphocyte ratio
OS: Overall survival
PD: Main pancreatic duct dilation
PNET: Pancreatic neuroendocrine tumor
ROC: Receiver operating characteristic
WHO: World Health Organization
